# A major QTL controlling apple skin russeting maps on the linkage group 12 of ‘Renetta Grigia di Torriana’

**DOI:** 10.1186/s12870-015-0507-4

**Published:** 2015-06-19

**Authors:** Luigi Falginella, Guido Cipriani, Corinne Monte, Roberto Gregori, Raffaele Testolin, Riccardo Velasco, Michela Troggio, Stefano Tartarini

**Affiliations:** Department of Agriculture and Environmental Sciences, University of Udine, Via delle Scienze 208, 33100 Udine, Italy; Department of Agricultural Sciences, University of Bologna, Via Fanin 44, 40127 Bologna, Italy; Research and Innovation Centre – Fondazione Edmund Mach – Department of Genomics and Biology of Fruit Crop, Via E. Mach 1, 38010 S Michele all’Adige TN, Italy

**Keywords:** *Malus x domestica*, Russet, Mapping, Quantitative Trait Locus (QTL), Single Nucleotide Polymorphism (SNP), Infinium**®** Illumina SNP chip

## Abstract

**Background:**

Russeting is a disorder developed by apple fruits that consists of cuticle cracking followed by the replacement of the epidermis by a corky layer that protects the fruit surface from water loss and pathogens. Although influenced by many environmental conditions and orchard management practices, russeting is under genetic control. The difficulty in classifying offspring and consequent variable segregation ratios have led several authors to conclude that more than one genetic determinant could be involved, although some evidence favours a major gene (*Ru*).

**Results:**

In this study we report the mapping of a major genetic russeting determinant on linkage group 12 of apple as inferred from the phenotypic observation in a segregating progeny derived from ‘Renetta Grigia di Torriana’, the construction of a 20 K Illumina SNP chip based genetic map, and QTL analysis. Recombination analysis in two mapping populations restricted the region of interest to approximately 400 Kb. Of the 58 genes predicted from the Golden Delicious sequence, a putative ABCG family transporter has been identified. Within a small set of russeted cultivars tested with markers of the region, only six showed the same haplotype of ‘Renetta Grigia di Torriana’.

**Conclusions:**

A major determinant (*Ru_RGT*) for russeting development putatively involved in cuticle organization is proposed as a candidate for controlling the trait. SNP and SSR markers tightly co-segregating with the *Ru_RGT* locus may assist the breeder selection. The observed segregations and the analysis of the ‘Renetta Grigia di Torriana’ haplotypic region in a panel of russeted and non-russeted cultivars may suggest the presence of other determinants for russeting in apple.

**Electronic supplementary material:**

The online version of this article (doi:10.1186/s12870-015-0507-4) contains supplementary material, which is available to authorized users.

## Background

Russeting is a common disorder that affects the peel of different organs (i.e. fruits and tubers) in several species such as potato, tomato, apple and pear [1–5]. The consumer perception of russeted fruits is quite different among species. For example, russeting in the pear is an important quality attribute of the fruit, while apple russeting is often considered negative. Great interest has been raised by apple clones that are less prone to russeting than the original cultivar, such as ‘Golden Delicious Smoothee®’ and ‘Golden Reinders®’ as compared with the original ‘Golden Delicious’ (GD) variety. In the past, russeting was not considered a defect since it was associated with increased aroma perception [4]. Interestingly, recent studies have demonstrated that suberized skin on russet varieties contains a peculiar class of chemicals that have been shown to have immunomodulatory activity, the triterpenes-caffeates [6]. The potential beneficial effect on human health may therefore give rise to renewed interest in russeted varieties. In apple, russeting predominantly occurs on the stalk or eye cavities, as patches scattered over the cheeks or covering the whole fruit [7]. Environmental conditions and growing practices can heavily influence russet formation. Several works have demonstrated that a range of abiotic and biotic agents may favour russeting outbreak such as prolonged periods of high levels of surface moisture and humidity [8–11], chemical applications [12, 13], mechanical wounding [14] and infection by pests or microorganisms [15–17].

In apple, russeting is thought to result from the formation of a plastic periderm in response to microcracking on stiff cuticle [4, 18, 19]. Following skin failure, the underlying cork cambium (phellogen) rapidly forms new cells (phellem) in order to replace the damaged epidermis and combat water losses. The depositing of suberized cell layers (periderm) thus gives rise to the typical brown and corky aspect of russeted apples [20–22]. The formation of microcracks is most likely due to cuticle incapability to keep pace with cortex growth, particularly during early developing stages, concomitantly with the fruit growth rate peaks [11, 23, 24]. Despite the progress in phenology and in the aetiology of apple russeting, the genetics underlying this phenomenon is still poorly understood. The genetic bases of apple russeting is supported by such evidences as (i) apple collections with cultivars that display considerable russeting variability, irrespective of growth conditions; (ii) the occurrence of russeted sport mutations of non-russeted cultivars, and (iii) the segregation of the character in controlled crosses. The occurrence of fruit sectorial chimeras and spontaneous/induced sport mutations have been reported in the literature, with either russet-free sports from russet susceptible cultivars or russeted sports from trees bearing fruits with no or little russet [4, 7, 25–27]. Examples of russeted bud sport mutants are ‘Siddington Russet’, ‘Norfolk Royal Russet’ and ‘Daligris’ that arose from ‘Galloway Pippin’, ‘Norfolk Royal’ [28] and ‘Pinova’ (USPP11601 P), respectively. Such evidence, plus the inheritance studies carried out on a number of crosses between genotypes with different russeting extents, provided the first evidence of the genetic control of the trait [7, 29, 30]. Alston and Watkins, considering the russeted sports and the segregation observed in the progeny of ‘Court Pendu Plat’ and ‘D’Arcy Spice’, stated that a simple genetic control (*Ru* gene) might be responsible for complete russeting [29, 30]. In contrast, a multi-factorial control for non-complete russeting has been claimed following the evaluation of offspring from combinations between partially russeted and either slightly to full russeted varieties [7, 30]. Segregation ratios observed in the progeny of the moderate russeted cultivars ‘Cox’s Orange Pippin’ suggested a major gene, the effect of which was modulated by further minor genes [30]. Conversely, polygenic control has been suggested in other crops such as potato and pear. In diploid potatoes, russet characteristics were found to be determined by the complementary action of three dominant genes inherited independently; a change in one of the three loci resulting in a transition to either direction [1,2]. In pear, a model based on two dominant genes (*R* and *I*) was thought to govern russeting in Japanese pear (*P. pyrifolia*): the *R* locus was responsible for russet development while the modifier *I* locus partially suppressed cork formation [5]. A complex control of partial russeting has also been inferred from genetic studies on the progeny of *P. communis* [31]. The objective of the present work was to investigate the genetic control of fruit russeting in apple. Towards this end, a dense genetic map was developed using a F1 segregating population obtained from the controlled cross between the full-russeted genotype ‘Renetta Grigia di Torriana’ (RGT) and GD, a cultivar characterized by slight to moderate russeting depending on environmental conditions.

## Results

### Phenotypic data assessment

**Fig. 1:**
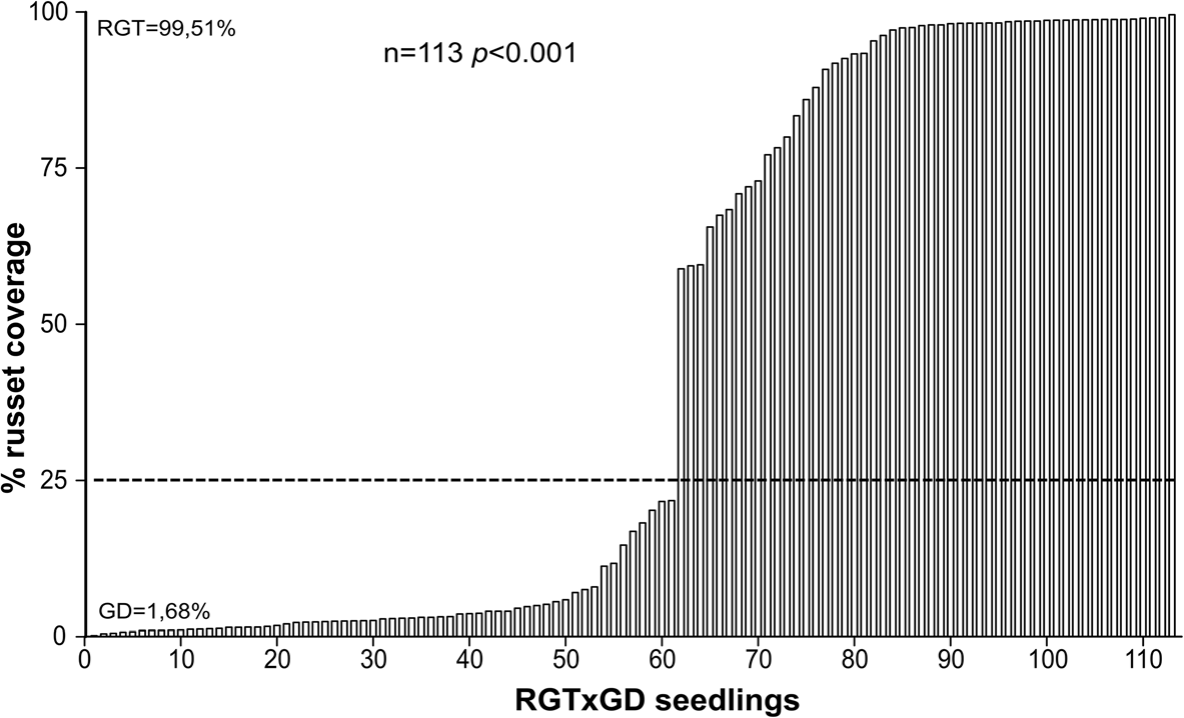
Distribution of F1 offspring from the RGTxGD cross based on the percentage of fruit russeting. The fruit russeting was measured in 2013 by means of digital image analysis. Plants were ordered on the basis of the average fruit russeting coverage. Dotted horizontal line indicates russeting percentage distinguishing between clean and russet genotypes as indicated by [30]. The average russeting coverage of the fruits of the two parental lines are reported beside the y axis. Number of observed individuals (n) and Shapiro-Wilks test significance for normality distribution are indicated on the top

Datasets of fruit skin russeting percentages recorded over four seasons from 2010 to 2013 consisted of RGTxGD populations ranging from 88 (2012) to 117 individuals (2010). Regardless of seasonal conditions, RGT and GD parents constantly showed russeting of 95-100 % and 0-10 %, respectively (data not shown). Correlation coefficients (*R*) of phenotypic data between years ranged from 0.96 to 0.99 (Table 1). Non-normal distribution of phenotypic data was statistically confirmed by the Shapiro-Wilks test (Table 1), which showed a significant deviation from normality (*p* < 0.001) occuring each year. The deviation from normality (*p* < 0.001) and a bi-modal distribution of data was also observed after images analysis in 2013 (Fig. 1). Data obtained from russet measurement from images strongly correlated with field data in the same year (Additional file 1). A sharp cut-off identified at about 25 % of russet coverage divided the progeny into two subsets (Fig. 1) and led to the hypothesis that a single major determinant might be responsible for the trait. The hypothesis was supported by the chi square test (Table 2) carried out on data organized according to the classification reported by [30] in which russet coverage of 25 % was fixed as the threshold for distinguishing clean (zero to slight russeting) from russeted apples (moderate to full russeting).

**Table 1:** Relationships between annual datasets of russeting field observations on the RGTxGD F1 population (Pearson’s coefficient of correlation), and Shapiro-Wilks test significance for normality distribution

Year	Observed genotypes	Pearson's correlation coefficient				Shapiro-Wilks test
		2010	2011	2012	2013	
2010	117	1				*p* < 0.001
2011	115	0.98	1			*p* < 0.001
2012	88	0.97	0.98	1		*p* < 0.001
2013	113	0.96	0.97	0.99	1	*p* < 0.001

**Table 2:** Segregation ratio of russet coverage observed on the RGTxGD F1 population following the classification proposed by [30]. Chi-square and p-values (one degree of freedom) are calculated under the assumption of a Mendelian 1:1 segregation ratio

Year	Clean	Russet	χ^2^ (1:1)	p-value
2010^a^	62	55	0.42	0.52
2011^a^	60	55	0.22	0.64
2012^a^	44	44	0.00	1.00
2013^a^	59	54	0.22	0.64
2013^b^	61	52	0.72	0.40

a = field observations

b = photos

### Genetic maps

**Fig. 2:**
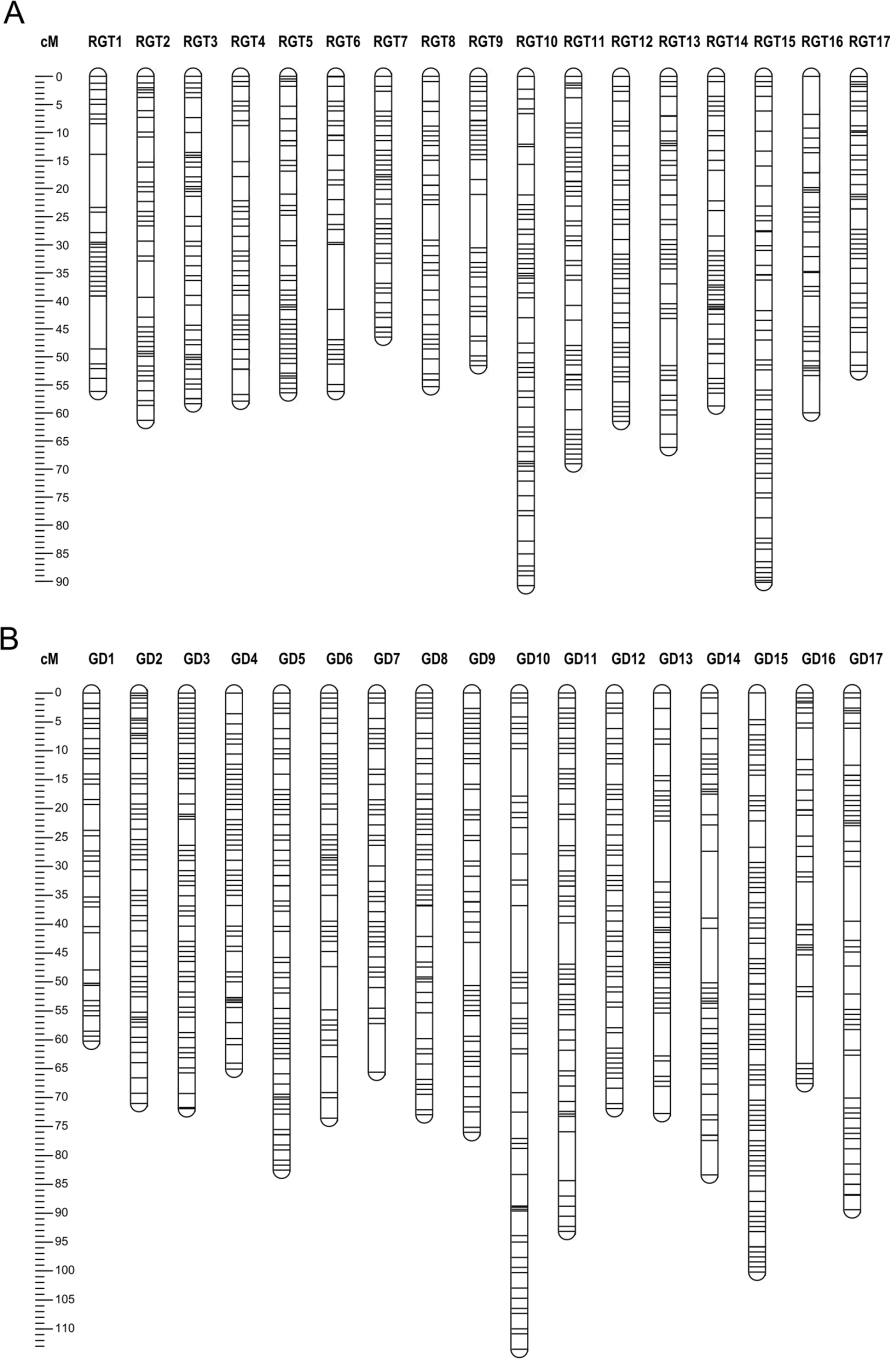
Distribution of unique co-segregating marker loci on the RGT **(a)** and GD **(b)** parental maps. Black bars represent either SNP or SSR markers. Linkage group (LG) number is indicated on the top of each LG. Genetic distance in cM is shown on the left ruler

The segregating population and parents were genotyped using the 20 K apple Infinium® SNP chip [32] produced by Illumina Inc. (San Diego, California, USA) and a set of microsatellites uniformly distributed across the 17 linkage groups (LGs) chosen accordingly to previous maps as described in the Methods. Array data mining identified 7,041 (39 % of the total 18,019 included in the array) polymorphic SNPs belonging to both the *abxaa* and *aaxab* segregating types that were retained to build maternal and paternal maps according to the double pseudo-test cross model [33]. The less informative *abxab* markers (n = 2,871 SNPs) were discarded as well as those monomorphic (n = 6,081) and those that failed or were difficult to score (n = 2,026). Of 188 tested SSRs, 160 were polymorphic and resulted in 170 map positions due to multiple loci. The RGT map consisted of 3,023 markers (2,870 SNPs and 153 SSRs) assembled into the expected 17 LGs, spanning 1,048 cM of genetic map distance, whereas GD map consisted of 4,663 (4,533 SNPs and 130 SSRs) markers grouped into the homologous 17 LGs, covering 1,331 cM. The number of markers mapped per LG varied from 122 of LG4 to 241 of LG10 in RGT parent, while in GD the range was from 133 of the LG7 to 527 markers of the LG15. Due to population size and the large portion of markers that co-segregated, markers were binned and only one SNP or SSR marker per locus was kept to obtain two abridged maps that consisted of 712 and 884 markers (Fig. 2), with a mean interval between adjacent markers of 1.47 cM and 1.51 cM for RGT and GD, respectively. Gaps between markers larger than 10 cM were found on the LG6 in RGT, and LGs 10, 13, 14, and 16 in the GD parent. Some regions with clear skewed marker segregation were found along some LGs of both RGT (5, 12 and 16) and GD (2, 6, 8, 14, 16 and 17). The full list of markers ordered by LGs, their segregation and skewedness is provided as additional material (Additional file 2).

### QTL analysis

#### QTL analysis of on field data

**Fig. 3:**
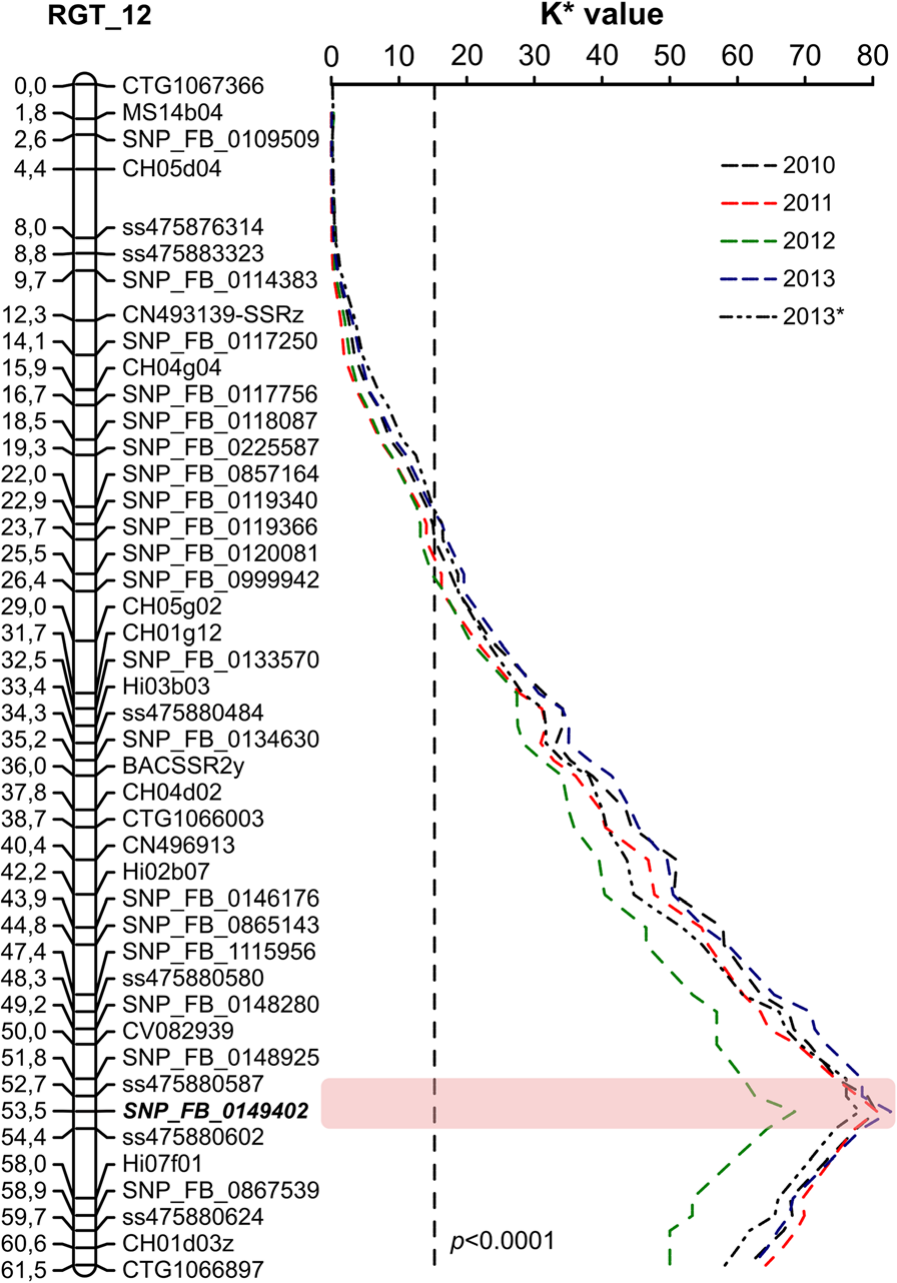
Quantitative trait locus (QTL) controlling the russeting identified on RGT LG12. Coloured dashed lines refer to K* values obtained after KW statistical test on four seasons transformed data from both field observations and digital photos analysis (2013*). The marker with the highest K* value across seasons and phenotyping methods is typed in bold and italics. Significance level at *p* < 0.0001 is represented by a vertical dashed line fixed at a K* value (one degree of freedom) of 16.2 as provided by the KW test on mean field data. The most significant QTL interval obtained through the MQM model is highlighted in pink

The Kruskal-Wallis (KW) statistical test showed a stable significant association between molecular markers and percentage of fruit russet coverage on LG12 in RGT (Fig. 3) that we named *Ru_RGT* locus according to [30]. The QTL peak co-segregated each year with the marker SNP_FB_0149402 and 6 further co-segregating SNP markers (Additional file 2) at approximately 53.5 cM from the top of the LG, a region that would refer to the contig MDC011810.169 of the apple v1.0 assembly. The K* values of the QTL peak associated with SNP_FB_0149402 ranged from 68.47 in 2012 to 82.66 (2013) (*p* < 0.0001) (Additional file 3). No additional QTLs were detected in the RGT genetic map, while the KW test identified further minor QTLs on LG2, 10, and 11 in the GD map (Additional file 3). As well as for the KW test, interval mapping (IM) carried out on condensed parental maps confirmed the presence of a strong and stable QTL on LG12 of RGT (data not shown). Marker SNP_FB_0149402 was constantly associated with the QTL peak for each of the four years, and explained from 71.9 % (2010) to 90.1 % (2012) of phenotypic variance. No further significant QTL was identified in the rest of the genome either in RGT or in GD by IM. A multiple QTL-mapping (MQM) analysis restricted the most significant QTL on LG12 to an interval of 2.6 cM, delimited by markers SNP_FB_0148925 (contig MDC009560.247) and ss475880602 (contig MDC021613.46) (Fig. 3). The region within these boundaries corresponded to a physical distance of about 1.3 Mbp on the apple reference genome [34].

#### QTL analysis on digital images

A QTL analysis was also performed on phenotypic data obtained in 2013 by digital photograph evaluation (Additional file 1). The KW test and IM confirmed the presence of a strong QTL in LG12 of RGT (K* = 77.98; LOD = 51.6), which was consistent with that identified with the field data (Additional file 3). The main QTL fell within the same interval as assessed by MQM on field data, and marker SNP_FB_0149402 showed the highest linkage. No further QTL was identified in any other LGs of RGT. In GD, the non-parametric test identified two minor QTLs: one on LG2 and another on LG10 (Additional file 3).

Two Genotype-Phenotype Incongruence (GPI) plants [35, 36] have been identified: plant 46 produced low russeted fruits but held the favorable allele for russet from RGT, while plant 105 had the alternative allele with the highly russeted fruits phenotype (around 50 % coverage). These two GPI plants were included in the primary QTL mapping.

### Fine mapping and candidate gene analysis

**Fig. 4:**
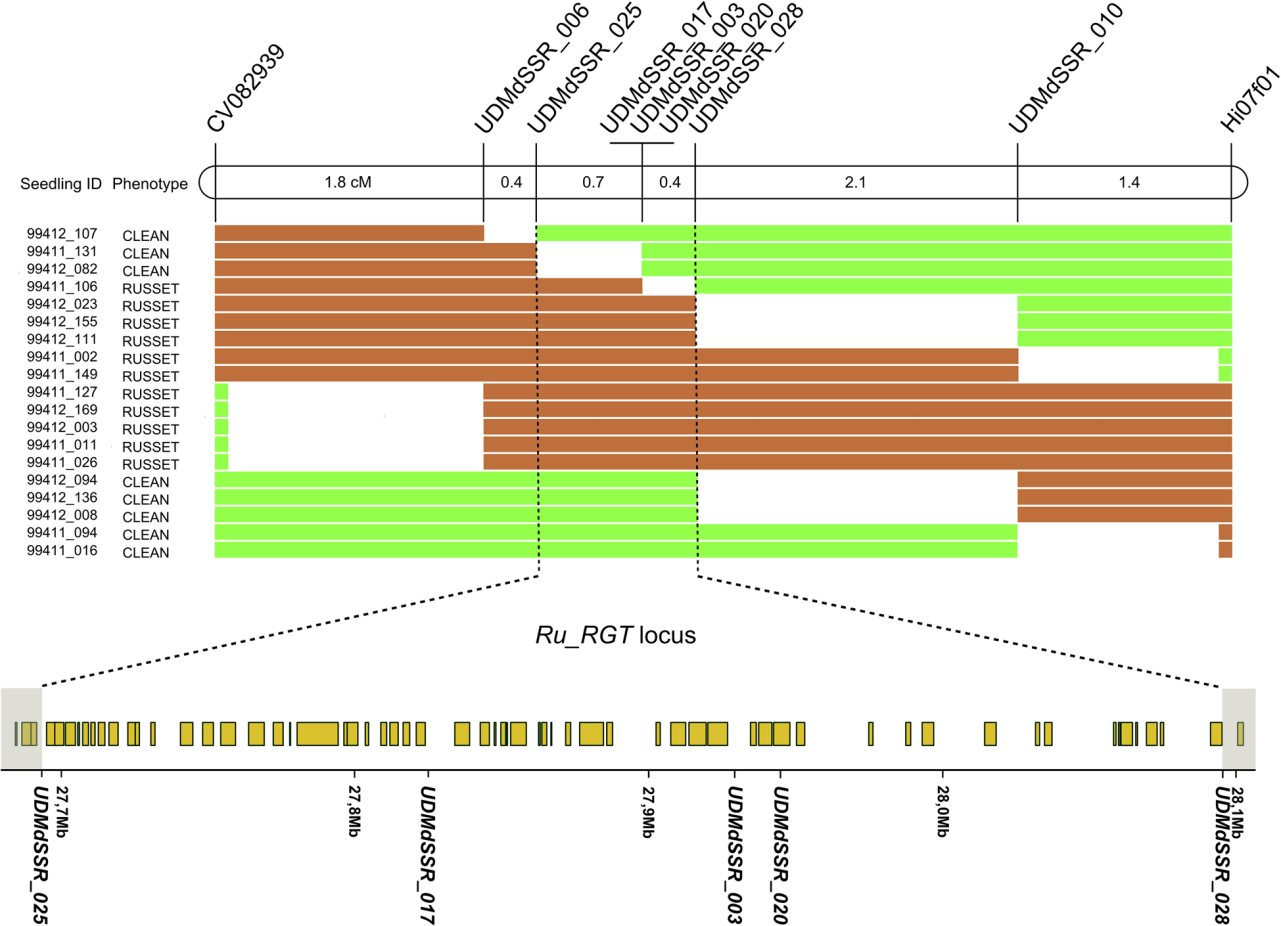
Fine mapping of the *Ru_RGT* locus on chromosome 12. Partial bottom region of the RGT LG12 is reported on the top as a horizontal bar. Genetic distances in cM between SSRs were calculated using recombination events occurred across 287 individuals from RGTxGD (code 99411) and RGTxGRH (code 99412) crosses. Recombinants between CV082939 and Hi07f01 SSRs are indicated on the left as well as the corresponding phenotype assessed for RGTxGD progeny according to [30]; brown bars represent the russeting haplotype, while green bars the alternative haplotypes. The restricted *Ru_RGT* locus is delimited by dotted vertical lines. Physical representation of the *Ru_RGT* locus is presented at the bottom according to the GDR Gbrowse; yellow rectangles represent annotated genes, while the position of SSR markers within the locus is indicated according to the Golden Delicious v1.0 assembly

To fine map the QTL on the LG12 of RGT, a set of microsatellite markers (coded as UDMdSSR) was newly developed from the sequence of the apple reference genome. Seven SSRs, physically close to the SNPs belonging to the *Ru_RGT* locus established by the MQM analysis, were found to be polymorphic in RGT (Additional file 4). The analysis was extended to the closest available external SSRs CV082939 and Hi07f01. The SSRs genetic position and co-association with SNPs were confirmed by genotyping the RGTxGD mapping population (Additional file 2). The *Ru_RGT* haplotype reconstruction was implemented by further testing these nine microsatellites on the 171 individuals of the RGTx‘GoldRush’ (GRH) cross. In a total of 287 seedlings, nineteen genotypes were found to recombine in the interval spanned by CV082939 and Hi07f01 markers (Fig. 4). The map order of the new SSRs was according to the v1.0 assembly and three recombinants enabled to fine map the *Ru_RGT* locus, between markers UDMdSSR_025 and UDMdSSR_028, to a physical interval of about 400 Kb in the reference genome sequence. Between these flanking markers, a cluster of three co-segregating SSR markers spanning about 150 kb (UDMdSSR_017, UDMdSSR_003, and UDMdSSR_020) was found and these markers were also co-segregating with the SNP_FB_0149402 (as found in the RGTxGD progeny).

The GD reference sequence at the QTL region was visualized in Gbrowse and the region directly downstream the UDMdSSR_025 marker (about 250 kb) resulted well-covered by a few long contigs while the remaining 150 kb towards the UDMdSSR_028 marker was rather fragmented with many short contigs and some gaps (Additional file 5). Within this region, a total of 58 genes were predicted by browsing the Genome Database for Rosaceae (GDR) (Additional file 6), most of which from the upstream region closer to the UDMdSSR_025 marker. Interestingly, a gene model (MDP0000200335) on contig MDC011810.169 showed best homology (e-121) with a plasma membrane-localized ATP-binding cassette half-transporter ABCG11 of *A. thaliana* (AT1G17840, Genbank no. NM_101647).

### Analysis of the russeting *Ru_RGT* haplotype in apple germplasm

**Fig. 5:**
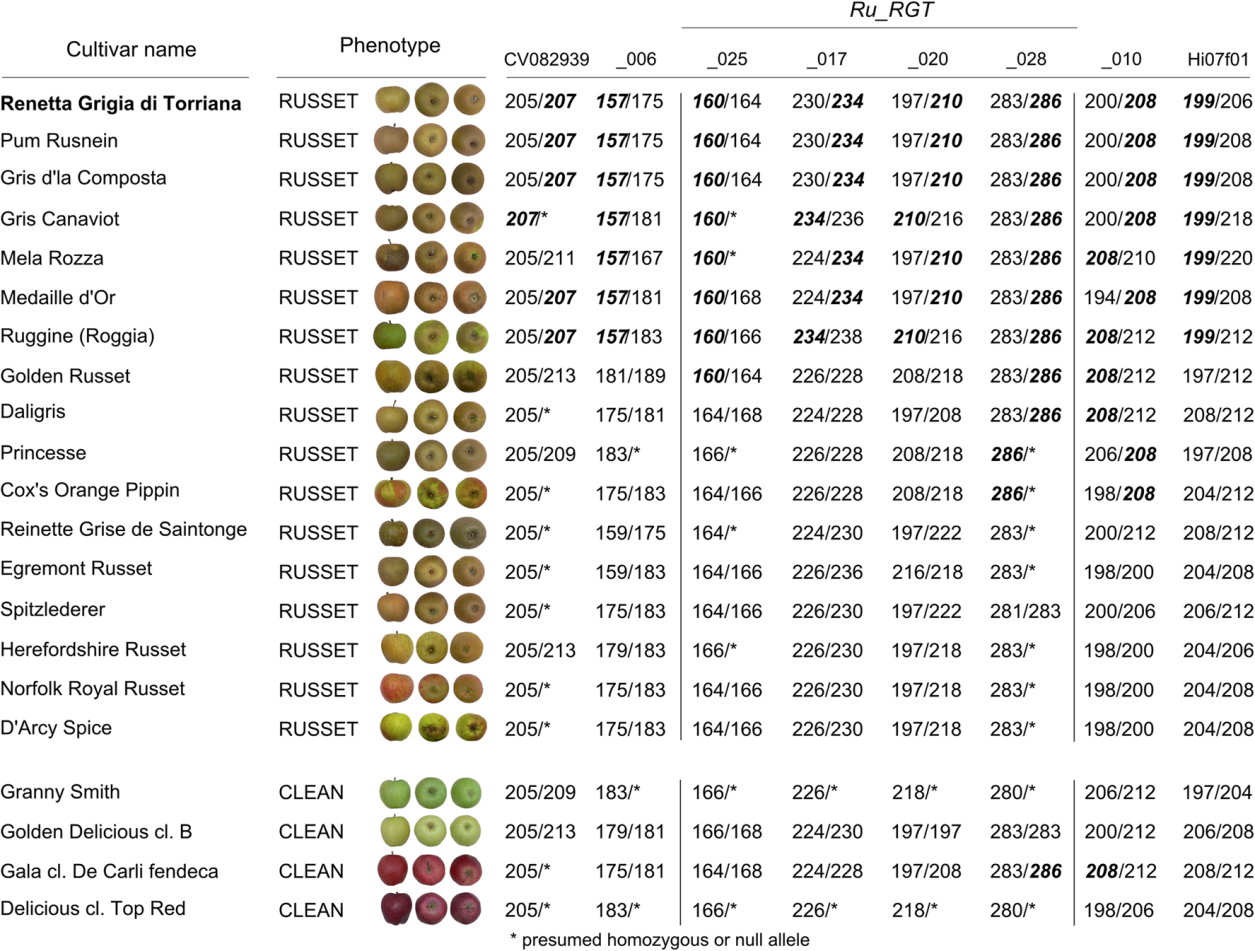
Haplotypes at the *Ru_RGT* locus on chromosome 12 in apple germplasm. A set of eight SSRs evenly distributed along the bottom region of the LG12, and containing the *Ru_RGT* locus was analysed in a group of 21 accessions of which 17 are reported as russeted according to [30] indications, while four controls have none or very little skin russeting. Markers are distributed according to map and physical positions from RGT and GD. Alleles coupled to skin russeting in RGT were highlighted in bold and italics. The length of UDMdSSR markers alleles includes the M13 tail. The restricted *Ru_RGT* locus is indicated by vertical lines

Seventeen russeted apple cultivars sorted out from apple germplasm and four non russeted (clean) cultivars were analysed both with eight SSR markers spanning 6.9 cM surrounding the *Ru_RGT* QTL region and 18 unlinked SSR markers to estimate their kinship. Seven russeted cultivars, RGT included, displayed the same haplotype associated to the *Ru_RGT* QTL at all markers of the region; the remaining ten russeted cultivars showed alternative alleles, with few exceptions for markers with more relaxed linkage to the *Ru_RGT* QTL, like UDMdSSR_25, UDMdSSR_028 and UDMdSSR_10, that occasionally showed the same alleles associated to the *Ru_RGT* haplotype (Fig. 5). Several of these alleles were also present in ‘Gala’, a non russeted cultivar. In the group of cultivars carrying the conserved *Ru_RGT* haplotype, only RGT and ‘Pum Rusnein’ showed a strong relatedness coefficient (r = 0.45), close to the expected value of 0.5 indicating a first-degree relationship (parent-offspring according to the analysis); the remaining cultivars of the group did not show remarkable kinship among them or with any other cultivar of the panel (Additional file 7). Conversely, the group of ten cultivars, that did not carry the *Ru_RGT* haplotype, showed extended relatedness (e.g. ‘Cox’s Orange Pippin’/‘Norfolk Royal Russet’, ‘Cox’s Orange Pippin’/‘Herefordshire Russet’, ‘Reinette Grise de Saintonge’/’D’Arcy Spice’, ‘Daligris/Norfolk Royal Russet’, ‘Egremont Russet’/’D’Arcy Spice’ and other pairs). Interestingly, several of these cultivars showed relatedness with GD and ‘Gala’, clean cultivars included in the panel only for the analysis of alleles alternative to the *Ru_RGT* haplotype (Additional file 7).

## Discussion

Although several studies on apple russeting aetiology enabled a better comprehension of the mechanical causes provoking this phenomenon, the genetics behind russeting was scarcely investigated according to the reviewed literature.

### Score classes and visual vs digital image analysis of russeting

The visual field russet phenotyping of the RGTxGD progeny across four seasons clearly showed that the segregation of russet skin coverage significantly fits the hypothesis of a major gene controlling the trait. The russeting threshold used by [29] to define clean and russet genotypes was adopted and was supported by our results, particularly when considering data from the analysis of digital photos. Data obtained through this method, although considering a limited number of sampled fruits and the limits of the bi-dimensional images, confirmed the results of visual scoring, but also appeared a more objective and precise method of analysis compared with visual scoring. This aspect was particularly relevant for confirming the russeting coverage threshold to distinguish between the two classes of clean and russet genotypes. Although in 2013 data from these two methods exhibited a high correlation, the digital scoring showed a clear discontinuity distribution at about 20-25 % skin coverage, consistently with [30] results.

### The 20 K Infinium® Illumina SNP chip for mapping and QTL analysis in apple

This is the first work reporting on the adoption of the 20 K Infinium**®** Illumina SNP chip for a QTL analysis survey. The QTL analysis revealed the great improvement in map construction afforded by the 20 K Infinium**®** Illumina SNP chip in terms of resolution and genome coverage in respect to the standard methods used so far (SSRs and AFLPs). The high density genetic maps obtained with the SNP array were integrated with known microsatellite markers for linkage group assignment. This dense map may prove very useful in future for correct landing in the apple genome sequence for SNP identification in specific genetic positions. Evidence reported in this paper strongly support the presence of a major QTL at the bottom of LG12 associated with apple skin russeting in the cultivar RGT and this determinant was named *Ru_RGT*. Despite the different genetic background, the observed segregation is in agreement with the model of a single gene (*Ru*) suggested by [30] for ‘D’Arcy Spice’. The *Ru_RGT* locus can explain most of the phenotypic variation observed for the trait but the presence of other genes that influence russet formation has to be postulated because of the differences observed in russet coverage both for plants carrying and for those not carrying the *Ru_RGT* gene. Several QTLs located on the lower portion of chromosome 12 were indicated as involved in resistance/tolerance to fungal and bacterial diseases [37–39] or in controlling fruit quality and phenology traits [40–44]. However, the LG12 has never been indicated before as the chromosome where russet controlling genes would lie, neither in apple nor in pear. Recently a large phenotyping/genotyping study on an apple training population sought to test the accuracy of genomic selection in predicting genomic breeding values, indicated that a SNP marker (NCBI db ss475876799) on LG1 had the highest effect on skin russet coverage, while at least three other QTLs, on LGs 9, 16 and 17 respectively, had a moderate effect [45]. This discrepancy could be due to the different genotypes analyzed, where these could carry genes with similar functions located in different chromosomes, considering the ancestral polyploidization of apple genome, but none of these chromosomes are homeologous to LG12 [34]. Mapping studies in *Pyrus*, an apple-related genus, identified two QTLs controlling fruit skin russeting in LG16 [46] and LG8 [47] and again both these LGs are non-homeologous with LG12 [48]. Lack of synteny between apple and pear for specific traits was also observed for fruit red skin color (MYB10) mapped on the non-homeologous LGs 9 and 4, in apple and pear, respectively [49, 50]. The reliability of the three minor QTLs detected in this study on LGs 2, 10 and 11 would require a further validation on a large progeny. This because they were clearly not fully reproducible among years and detected only by the KW analysis. Furthermore, none of these putative QTLs regions were known as involved in russeting in published studies.

### Fine mapping of the *Ru_RGT* locus and identification of a candidate gene

Since the KW test showed that the significant QTL on LG12 encompassed a large part of the LG at a significance level of *p* < 0.0001 (df = 1), an MQM analysis carried out on the condensed map permitted restriction of the locus to a corresponding 1.3 Mbp interval of the reference genome. Recombinants of the region from the two segregating populations meant the candidate region could be reduced to a physical interval of about 400 Kb, between the newly developed SSR markers UDMdSSR_025 and UDMdSSR_028. A search of genes potentially involved in fruit skin organization or active on peel related molecules biosynthesis was performed and among the 58 genes annotated within this region, the gene model MDP0000200335 was identified as a good candidate for russeting control. The BLAST search assessed against the TAIR protein database indicated a strong similarity between the apple gene and the *Arabidopsis thaliana* ATP-BINDING CASSETTE G11 (AtABCG11). The gene also known as DSO (DESPERADO), COF1 (CUTICULAR DEFECT AND ORGAN FUSION 1), or AtWBC11 (*A. thaliana* WHITE-BROWN COMPLEX HOMOLOG PROTEIN 11) was demonstrated to encode for a G sub-family ABC half-transporter protein involved in cuticle development [51–53]. The encoded protein is reported to be involved in cuticle development, cutin and wax secretion, particularly in reproductive organs [51–54]. Cuticle is a polymer that consists of a C_16_-C_18_ fatty acids cutin matrix embedding waxes to form a complex hydrophobic layer aimed to protect inner tissues from water loss, biotic/abiotic stresses, and to prevent post-genital organ fusion. The *Arabidopsis* ABCG11 protein localizes in the plasma membrane, where it forms functional homo and/or heterodimer complexes [55] in order to play its role as cutin and wax monomers transporter from the inside of epidermal cells to the extracellular matrix. Recent *RNA-seq* studies on the sand pear (*P. pyrifolia*) [56, 57] and apple [58] pericarp transcriptome showed several genes differentially expressed between green/waxy and russet mRNA libraries. In the Japanese pear, some ABC transporters involved in cuticular lipids precursors transport displayed transcriptional differences among russeted and non-russeted genotypes. Transcripts of the unigene GALR01022677, which showed a high similarity with *Arabidopsis* ABCG family transporters, were more abundant in green exocarp than in russeted skin, while conversely the gene GALR01018331 exhibited higher expression in the russet peel [57]. In apple, the comparison of bulk transcriptomic profiles from russeted and waxy genotypes, showed that gene model MDP0000200335 and its putative homeologous on LG4 (MDP0000248808) were greatly under-expressed in russeted cultivars at harvest time [58]. Though supporting the hypothesis of a principal role in russeting control unrolled by ABC transporters both in pear and apple, these data were obtained from 80 and 150 days old fruits, respectively, representing a single snapshot of exocarp transcriptome during fruit growth, without considering early development stages. Russeting occurs early in RGT fruits, concomitantly with the cell division phase and initial part of cell expansion phase during which a relative growth peak rate is normally observed in apple [59]. Although the predicted gene model MDP0000200335 might represent a strong candidate for russeting control, neither the role of other genes annotated within the *Ru_RGT* locus nor the presence of cultivar specific genes not shared with the reference genome can be excluded.

### Conservation of the haplotypic region in different russet cultivars

The molecular analysis in a panel of 21 apple cultivars, including full, moderate and non-russeted genotypes, revealed that the full RGT haplotype was carried only by 6/17 russeted cultivars. Some of them could have a common origin because they come from the Italian germplasm but only two of them (‘Gris Canaviot’ and ‘Pum Rusnein’) cluster close to RGT [60]. The close relationship between RGT and ‘Pum Rusnein’ was also confirmed by our kinship analysis. The lack of evident relatedness of the remaining cultivars of this group, that share the same *Ru_RGT* haplotype and must share in turn a common ancestor, could be explained by the fact that they could be several generations away from each other. The scenario offered by the haplotypes of the remaining cultivars, that do not share the RGT haplotype, appears rather complex. Some close relationships with GD were expected because GD is in the pedigree of both ‘Gala’ (=‘Kidd’s Orange Red’xGD) [61] and ‘Daligris’, being mutant of ‘Pinova’, (=‘Clivia’xGD). Furthermore, an involvement of GD in the unknown pedigree of ‘Herefordshire Russet’ can be postulated. The presence of alleles flanking the *Ru_RGT* gene in some cultivars could be explained through recombination that could have occurred in their pedigree. The fact that some of these alleles were found also in one of the non-russeted controls (cv. ‘Gala’) also suggest that some of these alleles could be rather common in apple germplasm. Other russet-controlling loci not present or not expressed in RGT could therefore be postulated to explain the absence of the whole *Ru_RGT* haplotype, or at least the alleles of the markers UDMdSSR_017 and UDMdSSR_020 most tightly linked to the locus in ten russeted genotypes. Duplicated loci controlling specific traits carried by different chromosomes are common in apple and this is due to apple polyploidization demonstrated by the recently published genome sequence [34].

## Conclusions

A major QTL controlling apple peel russeting on LG12 of the russet cultivar RGT is reported in our work. A fine mapping approach narrowed the locus approximately to a 400 Kb interval, according to the reference apple genome. Gene annotation in this region revealed a potential candidate for russeting control, an ABC transporter likely involved in cuticle organization. Further studies are however needed to confirm identification of the genetic determinant and its role in russeting control. Molecular markers closely linked to the *Ru_RGT* gene were developed to help marker assisted selection at least in RGT crosses, considering that several russet cultivars did not carry the allele suggested for the molecular selection.

## Methods

### Plant material

The QTL detection was performed on a F1 population of 116 individuals derived from the cross RGTxGD (clone B). RGT variety bears fully russeted fruits and is locally grown in the Piedmont region of northwestern Italy, while the widely grown GD shows slight to moderate susceptibility to russeting depending on environmental conditions. An additional segregating family (n = 171) from the cross RGTx‘GoldRush’ (GRH), a hybrid from the GDxPRI Co-op17 cross, was used to validate QTLs and for fine mapping. Progeny plants were coetaneous, grafted on ‘M.9’ dwarfing rootstocks and planted in single copy in the experimental farm of the Department of Agricultural Sciences of the University of Bologna (Italy) (44°32'25.5"N 11°23'12.7"E). Trees were trained at a spindle and sprayed following common practices avoiding any treatments aimed at russet control. A set of 21 apple varieties characterized by a range of russet extent from none to full was also analyzed; trees were kept at the experimental farms of the University of Bologna, of the University of Udine (Italy) (46°01'55.1"N 13°13'21.2"E), and at the repository of local germplasm of the Friuli Venezia-Giulia region (46°00'28.6"N 13°01'53.3"E).

### Skin russet phenotyping

All progeny plants of the RGTxGD progeny were evaluated in the field for skin russet coverage across four seasons (2010–2013), while the RGTxGRH plants were scored only in 2010, 2012 and 2013. The entire yield of each genotype was observed by two trained evaluators at harvest; fruit russet coverage was determined adopting a percentage scale ranging from 0 % (no russet) to 100 % (fully russeted). In 2013, skin russet coverage of RGTxGD family was also digitally assessed by means of a photographically based method. With this aim, six representative fruits from each tree were collected at harvest, and stored at 4 °C until the analysis. Two groups of three apples each were cut along the longitudinal and equatorial axis, respectively. The peel sides of the twelve halves together were photographed by a Nikon D40 digital camera (Nikon, Shinjuku, Tokyo, Japan) placed over the apples at a fixed distance, under controlled conditions of light and exposure. TIFF format images were subsequently processed using Adobe Photoshop v5.0 (Adobe Systems, San Jose, CA, USA). After scale determination and background removal, the total planar area of selected fruits halves was calculated, and finally the whole russet fraction was automatically isolated using the magic wand tool and then subtracted from the clean area. The distribution normality of raw phenotypic data in the RGTxGD population was evaluated using the Shapiro-Wilks test.

### DNA extraction and genotyping

Young leaflets of each genotype from segregating progenies and cultivars were collected in 2 ml microtubes and then freeze-dried for subsequent DNA isolation. Genomic DNA was extracted using the DNeasy Plant Mini Kit (Qiagen, Hilden, Germany) and quantified with the Nanodrop ND1000 spectrophotometer (Thermo Scientific, Waltham, MA, USA). Genotyping of the RGTxGD family was initially carried out testing a set of 188 SSR primers, preliminarily selected to uniformly cover all linkage groups according to the HiDRAS website [62] and published linkage maps [63, 64]. Forward primers were labelled at the 5’ end with 6-FAM or HEX dyes (Sigma-Aldrich, St.Louis, MO, USA). A preliminary PCR test on the genomic DNA of the parents plus a limited offspring subset was led to evaluate markers polymorphism between RGT and GD. The 10 μl PCR reaction contained 1X HotMaster™ *Taq* Buffer (5Prime, Hamburg, Germany), 0.25 mM dNTP, 0.2 μM of forward and reverse primers, 0.5 U of HotMaster™ *Taq* DNA Polymerase (5Prime, Hamburg, Germany) and 10 to 20 ng of template DNA. PCR steps consisted of 2 min of initial denaturation at 94 °C, followed by 30–35 cycles of 20 s denaturation at 94 °C, 20 s annealing at 56 °C, 30 s extension at 65 °C, and 15 min of final extension at 65 °C. Two μl of 1:80 sterile ddH_2_O PCR dilution was mixed with 7.98 μl of formamide and 0.02 μl of GeneScan 500 LIZ standard (Life Technologies, Grand Island, NY, USA). The mixture was denaturated at 95 °C for 2 min, kept on ice for 5 min, and then run on an ABI3730 DNA analyzer (Applied Biosystems, Foster City, CA, USA). Run data were analyzed using GeneMapper v 4.0 software (Applied Biosystems, Foster City, CA, USA). The analysis of a subset of 89 individuals from the segregating population was carried out by PCR multiplexing polymorphic markers between parents according to fluorescence and alleles size, using the Multiplex PCR Kit (Qiagen, Hilden, Germany) in accordance with the manufacturers’ instructions. Amplicons analysis was done as described above. Subsequently the population was genotyped using the 20 K Infinium® SNP array [32] recently developed within the frame of the European project Fruitbreedomics. Two hundreds nanograms of genomic DNA from the two parents and 116 individuals were analyzed following the standard Illumina protocol detailed in [65]. Genotyping data were analyzed using the Genotyping Module of the Genome Studio Data Analysis Software V2011.1 (Illumina Inc., San Diego, California, USA) with a GenCall threshold of 0.15. Development of new SSR markers (prefix UDMdSSR) for fine mapping was achieved via the web interfaces of WebSat [66] and Primer3 [67] softwares, using as template genome contig sequences from the *Malus* x *domestica* v 1.0 assembly [34] with the Gbrowse tool of the GDR [68]. A M13 primer tailing strategy was adopted to test the new SSRs, including a forward primer tailed with the universal M13 sequence (5’-tgtaaaacgacggccagt-3’) at the 5’ end, a normal reverse primer, and a M13 primer labelled with 6-FAM or HEX dyes. PCR reaction was prepared as described above, excepting for 0.08 μM tailed forward primer, 0.2 μM reverse primer and 0.4 μM fluorescent labelled M13 primer. The touch-down PCR amplification consisted of a 2 min initial denaturation step at 94 °C followed by 5 cycles of 20 s denaturation at 94 °C, 40 s of 1 °C decreasing annealing temperature every second cycle from 60 °C, 40 s of extension at 65 °C, and 25–30 cycles of 20 s denaturation at 94 °C, 40 s annealing at 55 °C, 45 s extension at 65 °C and the final 15 min extension at 65 °C. Fragments screening was assessed as previously described. Genotyping of germplasm was led adopting newly developed SSR as described above.

### RGT and GD genetic map construction and QTL mapping

The construction of parental linkage maps was carried out adopting the ‘two-way pseudo-testcross’ mapping strategy [33]. Microsatellites data were visually screened, while for SNP data Genome Studio genotype calls were automatically processed through an automated SNP filtering pipeline [32] so as to discard unreliable SNPs and filter markers with less than 5 % of missing data, and a GenTrain score lower than 0.4. Microsatellites and SNPs monomorphic in both parents were not considered as well as markers segregating in an *hkxhk* fashion. Fully informative SSRs markers (*abxcd* and *efxeg*) were reciprocally considered as homozygous in one parent and heterozygous in the other (backcross type). Molecular markers showing identical segregation patterns were merged in a single genetic bin and a single marker per bin was kept for maps construction. The linkage analysis was performed using JoinMap® version 4.1 [69], using the independence LOD grouping parameter with a LOD score higher than 10, and the Maximum Likelihood (ML) mapping algorithm for calculation of genetic distances with default parameters. Markers at fixed distances and equally distributed to cover the seventeen LGs were used to build framework parental maps for the subsequent interval mapping analysis. Grouping was led with the same parameters as described above. QTLs detection was performed with the MapQTL® v5.0 [70] using the non-parametric KW test. QTL analysis was performed on transformed data adopting the angular transformation formula *θ=arcsin*√*x*, where *θ* is the transformed value and *x* the observed proportion. Similarly to [43], QTLs were considered if at least four adjacent markers showed significant K* value at a *p*-value lower than 0.01. QTL validation was assessed through IM and MQM run on condensed maps due to restricted computing capacity, following same conditions proposed by [71].

### Fine mapping and candidate gene search

Screening of the two segregating populations for informative recombinants was led using microsatellite markers developed as described above co-localized with SNPs spanning the significant *Ru_RGT* locus previously determined through the MQM analysis in the RGTxGD mapping population. Further SSRs putatively localized within the predicted genomic region were also developed and tested in both populations. The genome interval established by recombination events observed in RGTxGD and RGTxGRH families was scanned for gene models using the GDR Gbrowse tool on the v1.0 apple genome assembly. To identify candidate genes for russeting control the putative function of annotated apple genes was declared as the best match of a BLAST similarity search against the *Arabidopsis* protein dataset.

### Evaluation of genetic relationships

A set of eighteen unlinked polymorphic microsatellites covering 17 chromosomes (Hi07f01, CV082939, CH02d08, CH05a04, CH02d11, CH01c06, CH04c07, CH01h02, CH03g07, CTG1065894, CH-Vf1, Hi07h02, CN872071, CH05d02, Hi04g05, CH03d07, COL, CH03a09) was chosen to genotype a set of 21 cultivars carrying fruits with a different degree of russeting coverage. These SSR data were used to perform a Kinship analysis using KINGROUP v2.0 [72]. The pairwise relatedness coefficients (r) and associated *p*-values were estimated for each possible pair groups of cultivars using the relatedness estimator from [73]. Likelihood ratios (LR) plus associated *p*-values were calculated for each relatedness relationship considering a claimed relationship category as the primary hypothesis (*H*_*1*_) versus the subsequent closest genealogical relationship or unrelated category as the null hypothesis (*H*_*0*_).

## Additional files

Additional file 1:
**Fruit russet phenotyping by digital images.** Example of fruit sections used for the acquisition of digital images (A), russeting extent measurement (B) and correlation between on field and photos phenotyping (C). Black bar in (B) represents 20 mm. Additional file 2:
**SNP and SSR markers segregating in the RGT and GD parents.** The analysis of the 20 K Infinium® SNP array resulted in the identification of 2,870 and 4,533 segregating markers in the RGT and GD parents, respectively. The alignment of the linkage groups with previously published maps was performed by the analysis of 153 (RGT) and 130 (GD) microsatellite markers in a subset of 89 individuals from the RGTxGD progeny. Markers are reported according to their map position. Datasets include the LG assignment, the map position of each marker, the SSR name (HiDRAS) and dbSNP (NCBI) accession code, the locus phase, the frequencies of the genotypes for each locus plus number of missing data, the associated chi-square test results for segregation according to the 1:1 Mendelian ratio and distortion significance levels. Last column included the cosegregant marker name for each predicted position. Additional file 3:
**List of significant QTLs identified for fruit russet coverage in the cross between RGT and GD.** The analysis was performed adopting the KW statistical test on *arcsin* transformed data. Significant QTLs according to the Methods chapter are reported for each year of observation and phenotyping evaluation. Markers with highest *K** value are indicated as well as their genetic position on the LG and the significance levels. Additional file 4:
**UDMdSSR microsatellite primer sequences.** The ‘forward’ and ‘reverse’ primer sequences are reported for each SSR as well as the name of the contig carrying each SSR in the reference v1.0 assembly within the GDR genome browser [68]. Additional file 5:
**Graphical representation of the** ***Ru_RGT*** **contigs from the v1.0 assembly in the GDR genome browser** **[** 68 **].** General view of the assembled region (A) and a more detailed figure with contig names and markers position (B). Additional file 6:
**List of candidate genes found in the** ***Ru_RGT*** **QTL region for skin russeting.** Identification model and physical positions in the reference genome of GD were retrieved from the v1.0 assembly in the GDR genome browser [68]. *A. thaliana* genes and their functional description are reported as given by the TAIR blast tool. Additional file 7:
**Kinship analysis of a 21 apple varieties set characterised by different extent of skin russeting.** Eighteen microsatellites were used to perform a kinship analysis using the KINGROUP v2.0 software. Statistically significant relationships among apple varieties are reported in (A), with corresponding relatedness coefficients (r) and associated *p*-values. Likelihood ratios for specific comparison between relationship categories are indicated in (B). Flags represent significance levels at *p* < 0.05 (*), *p* < 0.01 (**), and *p* < 0.001 (***).

## Abbreviations

AtABCG11: *Arabidopsis thaliana* ATP-BINDING CASSETTE G11
AtWBC11: *Arabidopsis thaliana* WHITE-BROWN COMPLEX HOMOLOG PROTEIN 11
COF1: CUTICULAR DEFECT AND ORGAN FUSION 1
DSO: DESPERADO
GD: Golden Delicious
GDR: Genome Database for *Rosaceae*
GPI: Genotype-Phenotype Incongruence
GRH: GoldRush
HiDRAS: High-quality Disease Resistant Apples for a Sustainable Agriculture
IM: Interval Mapping
KW: Kruskal-Wallis
LG: Linkage Group
LR: Likelihood Ratio
ML: Maximum Likelihood
MQM: Multiple QTL-mapping
NFC: National Fruit Collection
PCR: Polymerase Chain Reaction
QTL: Quantitative Trait Locus
RGT: Renetta Grigia di Torriana
SNP: Single Nucleotide Polymorphism
SSR: Simple Sequence Repeat
TAIR: The Arabidopsis Information Resource
