# Supplementary figures and images for: A major QTL controlling apple skin russeting maps on the linkage group 12 of ‘Renetta Grigia di Torriana’
Source: BMC Plant Biol. 2015 Jun 19;15:150. doi: 10.1186/s12870-015-0507-4 (PMC4472412; doi:10.1186/s12870-015-0507-4)

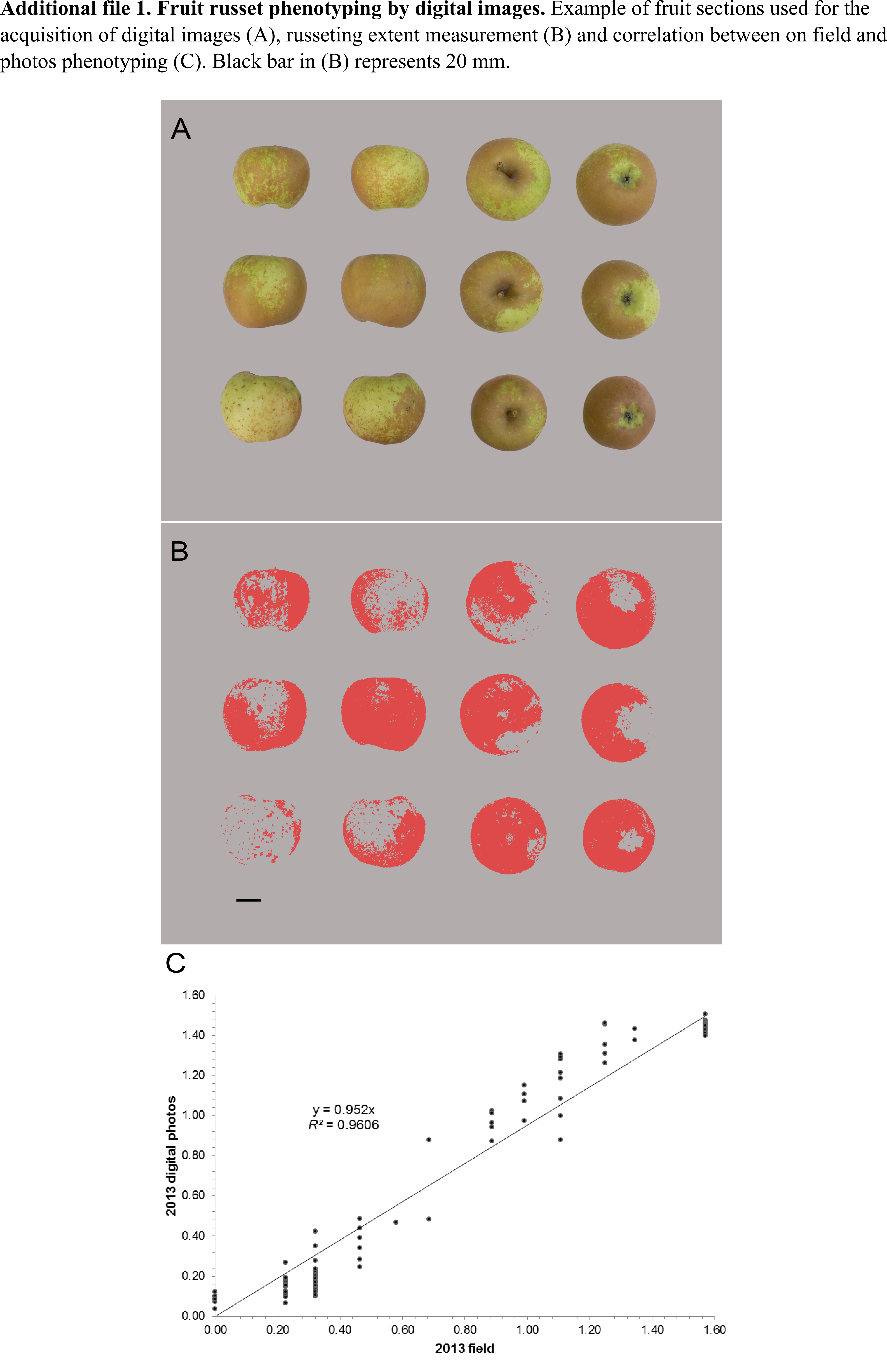

Supplement: Additional file 1: — Fruit russet phenotyping by digital images. Example of fruit sections used for the acquisition of digital images (A), russeting extent measurement (B) and correlation between on field and photos phenotyping (C). Black bar in (B) represents 20 mm. [file 12870_2015_507_MOESM1_ESM.png]

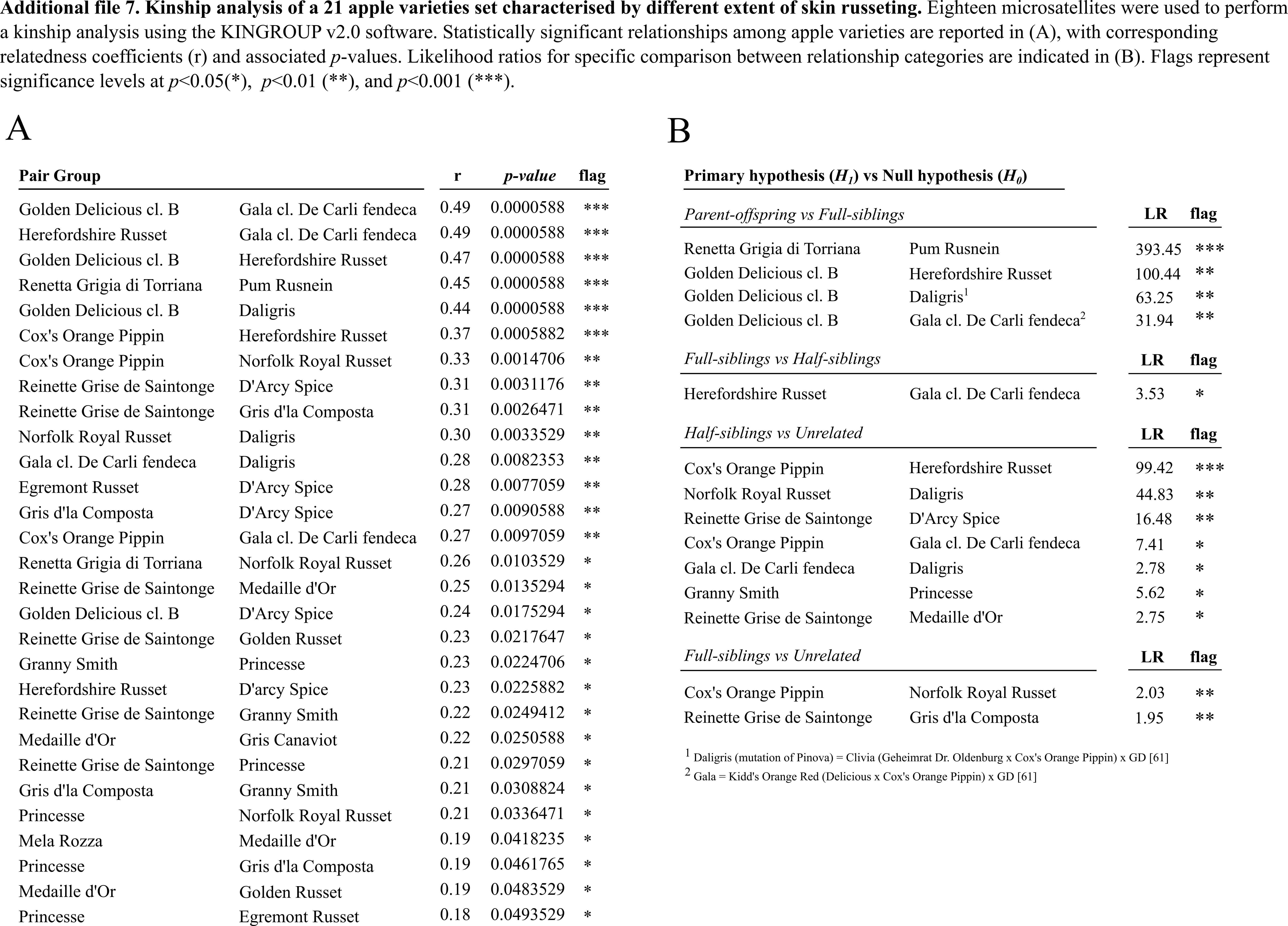

Supplement: Additional file 7: — Kinship analysis of a 21 apple varieties set characterised by different extent of skin russeting. Eighteen microsatellites were used to perform a kinship analysis using the KINGROUP v2.0 software. Statistically significant relationships among apple varieties are reported in (A), with corresponding relatedness coefficients (r) and associated p-values. Likelihood ratios for specific comparison between relationship categories are indicated in (B). Flags represent significance levels at p < 0.05 (*), p < 0.01 (**), and p < 0.001 (***). [file 12870_2015_507_MOESM7_ESM.png]
