# Supplementary material for: A major QTL controlling apple skin russeting maps on the linkage group 12 of ‘Renetta Grigia di Torriana’
Source: BMC Plant Biol. 2015 Jun 19;15:150. doi: 10.1186/s12870-015-0507-4 (PMC4472412; doi:10.1186/s12870-015-0507-4)
Supplement: Additional file 3: — List of significant QTLs identified for fruit russet coverage in the cross between RGT and GD. The analysis was performed adopting the KW statistical test on arcsin transformed data. Significant QTLs according to the Methods chapter are reported for each year of observation and phenotyping evaluation. Markers with highest K* value are indicated as well as their genetic position on the LG and the significance levels. [file 12870_2015_507_MOESM3_ESM.docx]

| **Additional file 3. List of significant QTLs identified for fruit russet coverage in the cross between RGT and GD.** The analysis was performed adopting the Kruskal-Wallis statistical test on *arcsin* transformed data. Significant QTLs according to the Methods chapter are reported for each year of observation and phenotyping evaluation. Markers with highest *K** value are indicated as well as their genetic position on the LG and the significance levels.   \| **Year** \| **LG** \| **Position (cM)** \| **Max *K** value** \| **Marker with highest *K** value** \| **Significance** \| \| --- \| --- \| --- \| --- \| --- \| --- \| \| 2010 \| RGT_12 \| 53.542 \| 80.50 \| SNP_FB_0149402 \| ******* \| \| 2011 \| RGT_12 \| 53.542 \| 80.64 \| SNP_FB_0149402 \| ******* \| \| 2012 \| RGT_12 \| 53.542 \| 68.47 \| SNP_FB_0149402 \| ******* \| \| 2013 \| RGT_12 \| 53.542 \| 82.66 \| SNP_FB_0149402 \| ******* \| \| 2013^*^ \| RGT_12 \| 53.542 \| 77.98 \| SNP_FB_0149402 \| ******* \| \| 2011 \| GD_02 \| 69.270 \| 11.29 \| SNP_FB_0504021 \| ***** \| \| 2013 \| GD_02 \| 69.270 \| 12.49 \| SNP_FB_0504021 \| ****** \| \| 2013^*^ \| GD_02 \| 69.270 \| 11.02 \| SNP_FB_0504021 \| ***** \| \| 2010 \| GD_10 \| 50.969 \| 11.14 \| GDsnp01267 \| ***** \| \| 2011 \| GD_10 \| 50.969 \| 9.43 \| GDsnp01267 \| **** \| \| 2013^*^ \| GD_10 \| 50.969 \| 8.99 \| GDsnp01267 \| **** \| \| 2010 \| GD_11 \| 48.683 \| 9.04 \| GDsnp10010 \| **** \| \| 2011 \| GD_11 \| 47.813 \| 9.82 \| SNP_FB_0078778 \| **** \| \| 2012 \| GD_11 \| 47.813 \| 8.83 \| SNP_FB_0078778 \| **** \| |  |  |  |
| --- | --- | --- | --- | --- | --- | --- | --- | --- | --- | --- | --- | --- | --- | --- | --- | --- | --- | --- | --- | --- | --- | --- | --- | --- | --- | --- | --- | --- | --- | --- | --- | --- | --- | --- | --- | --- | --- | --- | --- | --- | --- | --- | --- | --- | --- | --- | --- | --- | --- | --- | --- | --- | --- | --- | --- | --- | --- | --- | --- | --- | --- | --- | --- | --- | --- | --- | --- | --- | --- | --- | --- | --- | --- | --- | --- | --- | --- | --- | --- | --- | --- | --- | --- | --- | --- | --- | --- | --- | --- | --- | --- | --- | --- |
| Significance levels: *0.1, **0.5, ***0.01, ****0.005, *****0.001, ******0.0005, *******0.0001 | | |  |
| ^*^=photos | |  |  |
