# Supplementary material for: A major QTL controlling apple skin russeting maps on the linkage group 12 of ‘Renetta Grigia di Torriana’
Source: BMC Plant Biol. 2015 Jun 19;15:150. doi: 10.1186/s12870-015-0507-4 (PMC4472412; doi:10.1186/s12870-015-0507-4)
Supplement: Additional file 4: — UDMdSSR microsatellite primer sequences. The ‘forward’ and ‘reverse’ primer sequences are reported for each SSR as well as the name of the contig carrying each SSR in the reference v1.0 assembly within the GDR genome browser [68]. [file 12870_2015_507_MOESM4_ESM.docx]

**Additional file 4. UDMdSSR microsatellite primer sequences.** The ‘forward’ and ‘reverse’ primer sequences are reported for each SSR as well as the name of the contig carrying each SSR in the reference v1.0 assembly within the GDR genome browser [68].

| Primer name | Forward primer (5’-3’) | Reverse primer (5’-3’) | Contig v1.0 |
| --- | --- | --- | --- |
| UDMdSSR_003 | TCGTAATGAGCTGGGATACACA | GACCTTTTGTGGTCAGGGAGTA | MDC011810.169 |
| UDMdSSR_006 | ACAACGAACAGGGAATGCTA | TGTTGATTCACCCATGGAATTGT | MDC014145.337 |
| UDMdSSR_010 | ACATACCAGCGAAGAGAGGCTA | AAAAGAAAACCCTACAACCCGT | MDC011802.265 |
| UDMdSSR_017 | GTACCTGAGAGAGAAAGCGAGC | ACTCCAGAAAAGGAAACATCCA | MDC020320.144 |
| UDMdSSR_020 | TCGTCTTCTTGCTCTTACCTCC | AAAACCCTAGCATTCAGTCAGC | MDC011810.169 |
| UDMdSSR_025 | AGAAGACTAAAATGCCTCTGCG | TATTGCTAACGATGTGGAAACG | MDC006184.101 |
| UDMdSSR_028 | ACTCCCCTGAAGAACACAATCA | TACTCCATCCATCCAGAAGCTC | MDC015856.337 |
