# Supplementary material for: A major QTL controlling apple skin russeting maps on the linkage group 12 of ‘Renetta Grigia di Torriana’
Source: BMC Plant Biol. 2015 Jun 19;15:150. doi: 10.1186/s12870-015-0507-4 (PMC4472412; doi:10.1186/s12870-015-0507-4)
Supplement: Additional file 5: — Graphical representation of the Ru_RGT contigs from the v1.0 assembly in the GDR genome browser [ 68 ]. General view of the assembled region (A) and a more detailed figure with contig names and markers position (B). [file 12870_2015_507_MOESM5_ESM.pptx]

## Slide 1
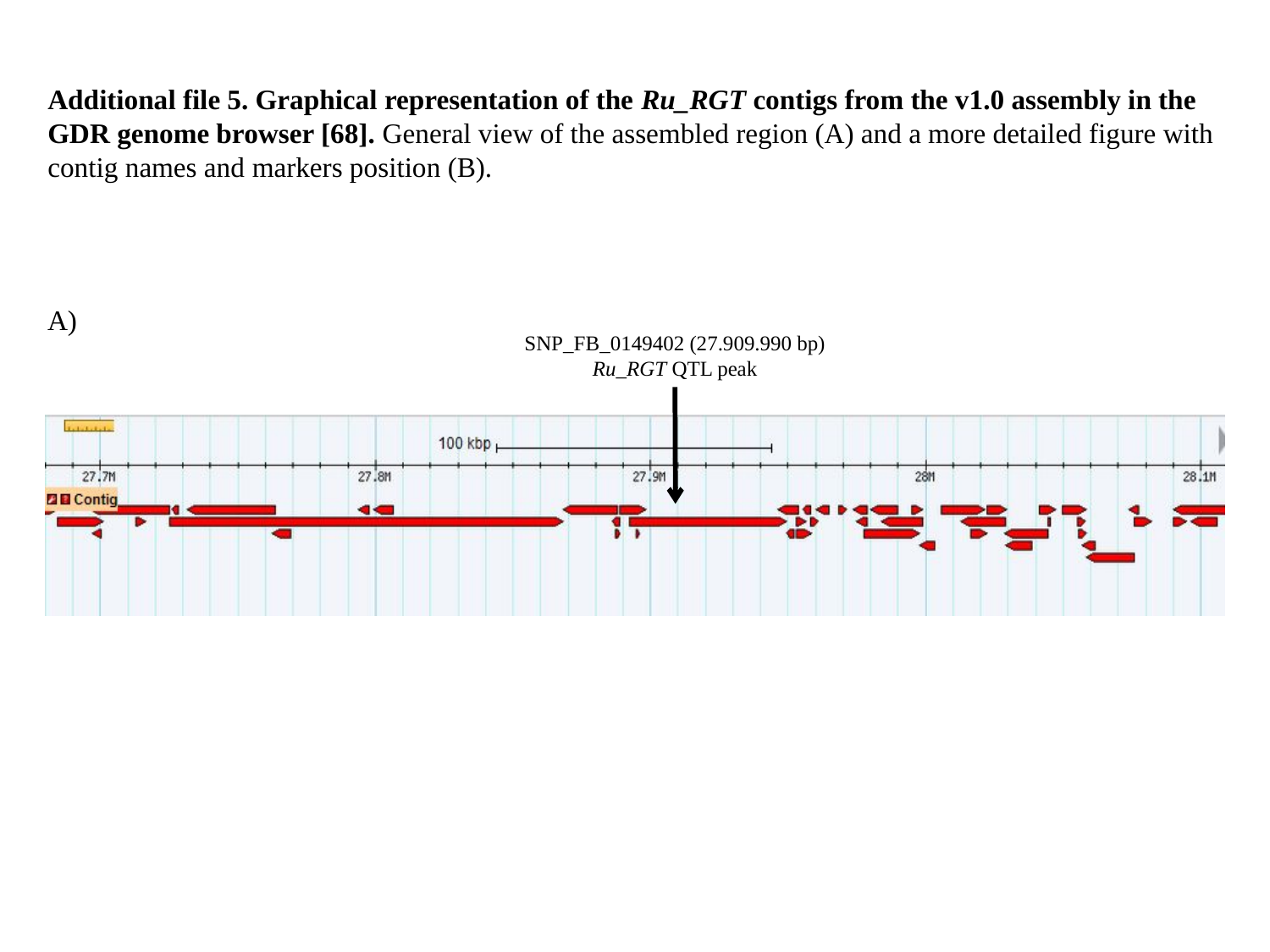

Additional file 5. Graphical representation of the Ru_RGT contigs from the v1.0 assembly in the GDR genome browser [68]. General view of the assembled region (A) and a more detailed figure with contig names and markers position (B).
A)
SNP_FB_0149402 (27.909.990 bp)
Ru_RGT QTL peak

## Slide 2
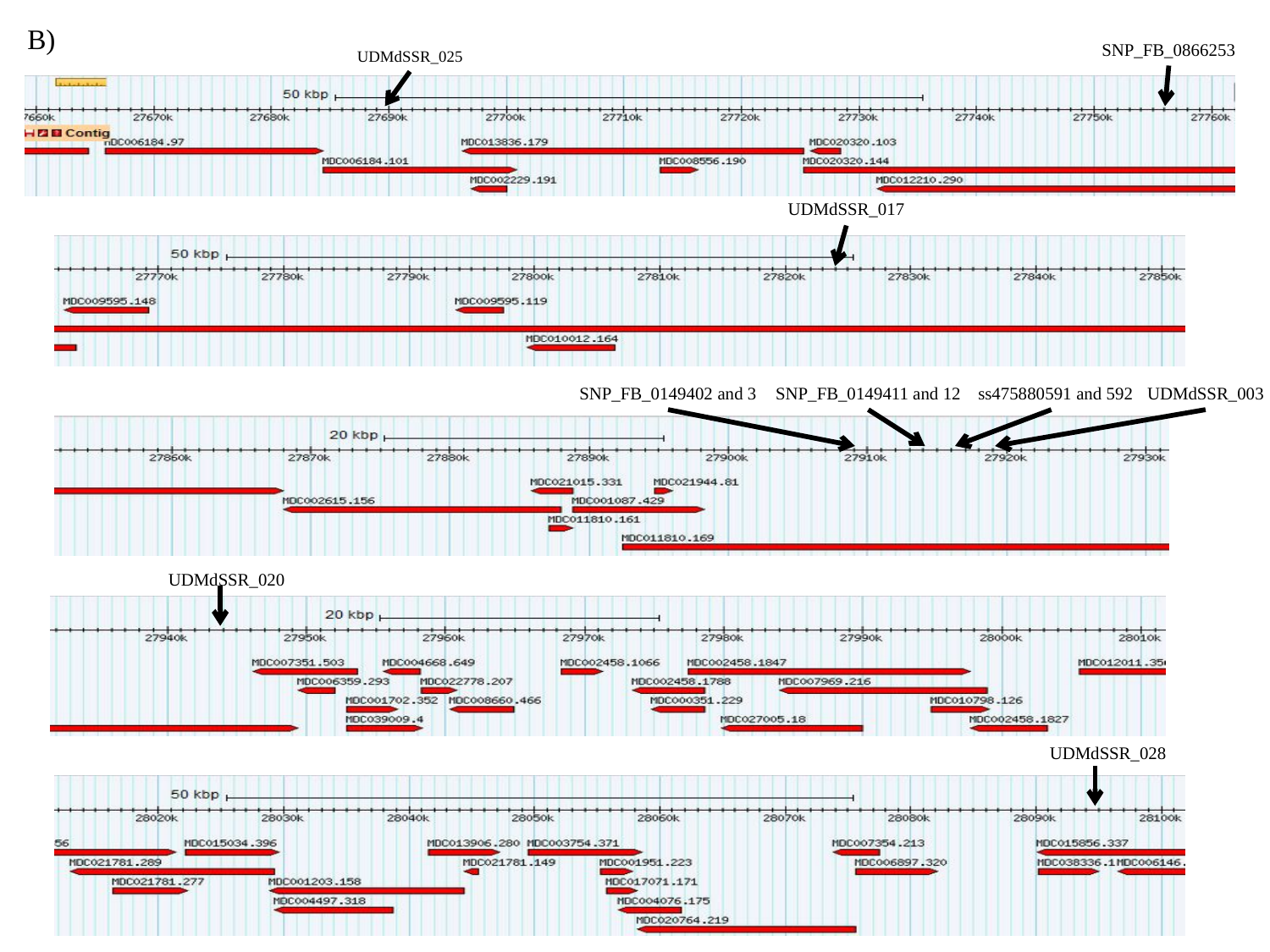

B)
SNP_FB_0866253
UDMdSSR_025
UDMdSSR_017
SNP_FB_0149402 and 3
SNP_FB_0149411 and 12
 ss475880591 and 592
UDMdSSR_003
UDMdSSR_020
UDMdSSR_028
